# Reinforcement Learning to Improve Image-Guidance of Ablation Therapy for Atrial Fibrillation

**DOI:** 10.3389/fphys.2021.733139

**Published:** 2021-08-25

**Authors:** Laila Muizniece, Adrian Bertagnoli, Ahmed Qureshi, Aya Zeidan, Aditi Roy, Marica Muffoletto, Oleg Aslanidi

**Affiliations:** ^1^School of Biomedical Engineering & Imaging Sciences, King’s College London, London, United Kingdom; ^2^Department of Biomedical Engineering, ETH Zürich, Zürich, Switzerland; ^3^Department of Computer Science, University of Oxford, Oxford, United Kingdom

**Keywords:** atrial fibrillation, catheter ablation, patient imaging, reinforcement learning, deep learning

## Abstract

Atrial fibrillation (AF) is the most common cardiac arrhythmia and currently affects more than 650,000 people in the United Kingdom alone. Catheter ablation (CA) is the only AF treatment with a long-term curative effect as it involves destroying arrhythmogenic tissue in the atria. However, its success rate is suboptimal, approximately 50% after a 2-year follow-up, and this high AF recurrence rate warrants significant improvements. Image-guidance of CA procedures have shown clinical promise, enabling the identification of key patient anatomical and pathological (such as fibrosis) features of atrial tissue, which require ablation. However, the latter approach still suffers from a lack of functional information and the need to interpret structures in the images by a clinician. Deep learning plays an increasingly important role in biomedicine, facilitating efficient diagnosis and treatment of clinical problems. This study applies deep reinforcement learning in combination with patient imaging (to provide structural information of the atria) and image-based modelling (to provide functional information) to design patient-specific CA strategies to guide clinicians and improve treatment success rates. To achieve this, patient-specific 2D left atrial (LA) models were derived from late-gadolinium enhancement (LGE) MRI scans of AF patients and were used to simulate patient-specific AF scenarios. Then a reinforcement Q-learning algorithm was created, where an ablating agent moved around the 2D LA, applying CA lesions to terminate AF and learning through feedback imposed by a reward policy. The agent achieved 84% success rate in terminating AF during training and 72% success rate in testing. Finally, AF recurrence rate was measured by attempting to re-initiate AF in the 2D atrial models after CA with 11% recurrence showing a great improvement on the existing therapies. Thus, reinforcement Q-learning algorithms can predict successful CA strategies from patient MRI data and help to improve the patient-specific guidance of CA therapy.

## Introduction

Atrial fibrillation (AF) is one of the most common cardiac arrhythmias, affecting about 1–1.5% of the general population with prevalence predicted to double by 2050 (Lip et al., 2007). Currently, the first-line treatment for AF is antiarrhythmic drug therapy, which can restore and maintain sinus rhythm (Zimetbaum, 2012). However, it has limited efficacy and can cause significant toxicity to organs outside the heart (Pollak, 1999). Catheter ablation (CA) is being increasingly used as a first-line treatment for AF with clinical trials demonstrating its superiority over antiarrhythmic drugs (Bunch and Michael, 2015). CA therapy is typically performed by the delivery of radiofrequency energy through a catheter which creates non-conductive lesions and thus, electrically isolates abnormal arrhythmogenic tissue from the rest of the heart.

AF is initiated by electrical triggers outside of the sinus node, typically near the pulmonary veins (PVs) – hence, pulmonary vein isolation (PVI) has become one of the cornerstones of CA (Bunch and Michael, 2015). However, a crucial issue concerning PVI and other ablation strategies is the high recurrence rate of AF post ablation (Jiang et al., 2014). This is often caused by PV reconnection post-ablation, which can occur in 94% of cases (Bunch and Michael, 2015). Moreover, multiple clinical trials have reported arrhythmia-free survival of only 50–75% at 1-year post ablation, with the highest recurrence rates associated with persistent AF cases (Kirchhof and Calkins, 2017) characterised by the presence of new AF triggers and drivers outside of the PVs. The latter have been strongly linked with atrial fibrosis (Nattel, 2016).

Fibrosis promotes AF *via* excessive collagen deposition in atrial tissue, which provides slow-conductive substrate for re-entrant drivers (rotors; Everett and Olgin, 2007; Boyle et al., 2019). Late gadolinium enhancement (LGE) MRI has proved to be an effective tool for non-invasive fibrosis quantification in AF patients, providing important information on spatial distributions of atrial fibrosis (Platonov, 2017). The availability of such data has also led to the development of patient-specific atrial models that link fibrosis distributions with the dynamics of rotors sustaining AF (McDowell et al., 2012; Boyle et al., 2019; Roy et al., 2020).

Recently, patient imaging data and image-based models are increasingly used in combination with novel artificial intelligence (AI) algorithms (Davenport and Kalakota, 2019), specifically to understand the mechanisms of AF and improve CA therapy (Lozoya et al., 2019; Feeny et al., 2020). Deep learning in particular is becoming widely used in applications such as image segmentation and patient classification (Yang et al., 2020). A promising, but less explored area of AI is Reinforcement Learning, where an algorithm learns based on a reward structure, similar to how a child learns by receiving rewards and penalties (Qiang and Zhongli, 2011).

Reinforcement Learning operates by allowing a free-moving agent to explore and interact with a given environment. The agent learns not from a predefined set of rules, but rather from the consequence of the actions it takes. This provides a perfect analogy with an ablation procedure, where a catheter moves in an environment of a patient atrial image (or an image-based model), and the reward comes in the form of a successful procedure, whereas failure to treat AF is a natural penalty; optimisation of the procedure comes from a large number of trials. Thus, the Q-learning process is similar to a cardiologist performing multiple image-guided ablation procedures on different patients and learning to apply the most suitable lesions in each case.

This study uses Reinforcement Q-learning algorithms to predict patient-specific CA strategies on a set of LGE-MRI based atrial models. These models include main structural features of the left atrium (LA), such as PVs, and employ advanced image-processing techniques to represent patient-specific fibrosis distributions and computational modelling to simulate AF scenarios.

## Materials and Methods

### Image-Based 2D Atrial Models

**Figure 1:**
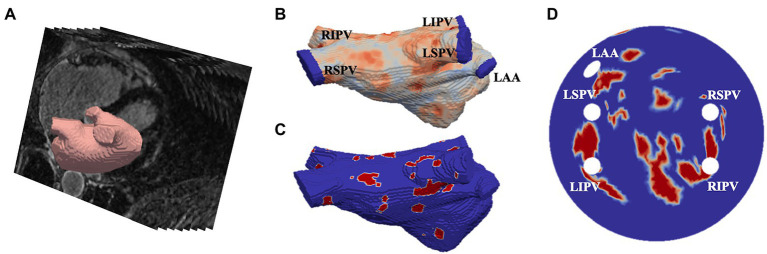
Generating image-based 2D left atrial tissues. **(A)** LGE-MR image (greyscale) with segmented LA (red). **(B)** The 3D LA with LGE-MRI intensity distribution and the PVs and LA appendage (LAA) clipped. **(C)** The thresholded 3D LA with fibrosis in red and healthy tissue in blue. **(D)** The LA unwrapped onto a standardised 2D disk, with fixed four PVs and the LAA and fibrotic areas mapped. PVs and LAA are labelled in **(B,D)**.

LGE-MRI data was acquired from two different sources. First, 86 scans were obtained from the 2018 STACOM segmentation challenge (Xiong et al., 2021), with resolution of 0.625×0.625×0.625mm^3^, and corresponding segmentations of the LA, and the second dataset was acquired from St Thomas’ Hospital (Chubb et al., 2018) from 18 AF patients, comprising of an additional 36 LGE-MRI images with a resolution of 1.3×1.3×4.0mm^3^, reconstructed to 0.94×0.94×2.0mm^3^. The patient images were processed in CemrgApp (Razeghi et al., 2020) using the scar quantification pipeline to first produce patient-specific 3D LA geometries with raw LGE intensity distributions. Then, the image intensity ratio thresholding technique was applied to clearly differentiate between fibrotic regions and healthy tissue (Roy et al., 2018). The resulting 3D LA dataset was then fed into an existing algorithm which unwrapped to a standardised 2D LA disk (Williams et al., 2017). The workflow of the image-based 2D LA tissue model generation is shown in Figure 1.

The final 2D LA models were used to simulate patient-specific AF scenarios, as described previously (Muffoletto et al., 2021). The monodomain equations were combined with the ionic Fenton-Karma equations (Roy et al., 2018) and solved on the 2D LA disks using the forward Euler method, using a temporal discretisation of 0.05ms and a spatial discretisation of 0.3mm. Zero-flux boundary conditions were implemented at the outer boundary of the disks and around the PVs. To model the slow-conducting properties of fibrosis, the monodomain diffusion coefficient was reduced from 0.05mm^2^/s in healthy tissue to 0.0075mm^2^/s in fibrotic patches.

**Figure 2:**
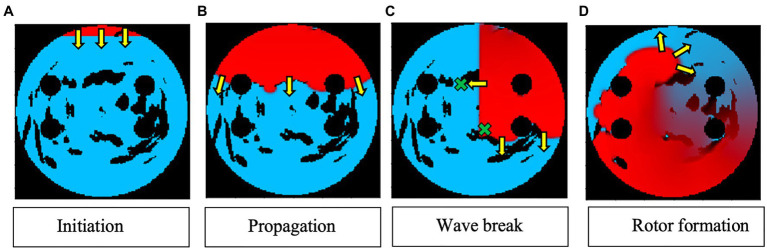
Rotor initialisation in a 2D LA tissue model. Voltage distributions in consecutive moments of time are shown in **(A)**–**(D)**, with red corresponding to high voltage, blue to the resting potential, and black to the PVs and area of fibrosis; yellow arrows show the directions of wave propagation. Green crosses show the locations of rotor initiation in two AF scenarios: a rotor illustrated in this figure corresponds to the lower right location, as seen in **(C)**.

Two AF scenarios were simulated for each LA model, each sustained by a rotor initiated in the tissue using a cross-field protocol, as shown in Figure 2. One rotor was initiated below the left superior PV (to initiate a rotor around the LSPV), while another was initiated between the inferior PVs (to initiate a free moving rotor). The distance between the two rotor initiation points was fixed at ~2.5cm. In the example shown in Figure 2, a plane wave is initiated at the top of the tissue and travels down two-thirds of the tissue, where the voltage on the left side of the tissue is then set to zero, initiating one rotor. The 2D LA models with simulated AF provided the environment for the Q-learning algorithm (see sections “Reinforcement Q-Learning Algorithm and Q-Learning Reward Structure” below). The same two AF scenarios (initial rotor locations) were used in both testing and training.

Once training and testing were completed, the successfully ablated tissue models were tested for rotor recurrence by attempting to initiate rotors in four different locations spread through the 2D LA tissue (resulting in simulation Scenarios 1, 2, 3, and 4). This was done to see whether the ablation strategy that was successful in termination of rotors would also work in preventing the emergence of new rotors. Recurrence testing helps to evaluate the long-term success due to the issue with AF reoccurrence after current ablation strategies.

### Reinforcement Q-Learning Algorithm

Q-Learning is initiated with a blank Q-table that assigns a value to each possible state to find the optimal policy for a given reward structure and therefore to maximise cumulative reward. The values in the table need to be enumerated by doing an extensive search over the action state space and recording, which combinations lead to positive or negative rewards. The best path to take in the Q-Learning process is mathematically described by Bellman’s Optimality equation (Moni, 2021):


$$V\left(s\right)=max_{a}\left(R\left(s,a\right)+\gamma V\left(s^{\prime }\right)\right)$$

Here, *s* is a particular state, *a* is the action, *s*′ is the state to which the agent moves to, *γ* is the discount factor, *R*(*s*,*a*) is the reward function, which takes a state *s* and action *a* and outputs a reward value, and *V*(*s*) is the value of a total reward for a particular state. This formula allows the agent to choose the path with the highest reward.

**Figure 3:**
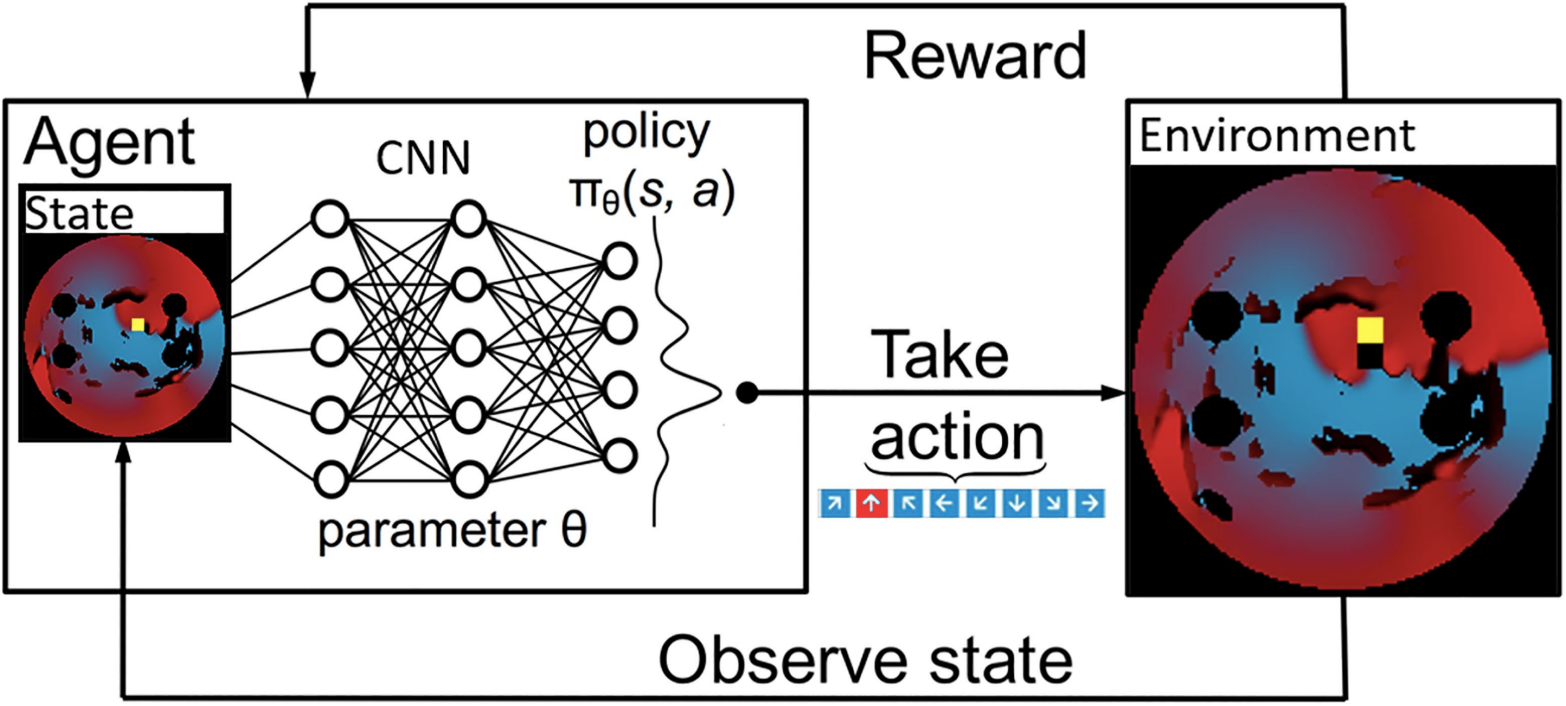
A schematic overview of how a deep Q-Learning network functions. Initially a state is observed, which is then passed through a CNN. The output of this network maps to the predicted Q values of all eight actions available. From here the action is chosen *via* a ɛ-greedy action selection policy. One notable addition to the implementation used in this paper is there are two copies of the network to ensure stability.

The 2D LA tissue model and simulated AF were used as an input for the Q-learning algorithm. Specifically, the agent’s environment is set to be the combination of the 2D diffusion matrix (the diffusion coefficients assigned per pixel of the 2D tissue), the simulated voltage at every point in the 2D tissue and, the values of activation variables at those same space–time points. These variables present different structural and functional properties of the same 2D LA tissue model that was used as the environment for the ablating agent. Figure 3 shows the most relevant part of the environment: 2D LA tissue structure with the voltage distribution in the form of a re-entrant wave, and the agent moving through this structure trying to terminate re-entry.

These variables were updated within an episode loop, which ran one different 2D LA tissue every episode, restarting the simulation with a new 2D tissue when a termination condition was reached. The LA tissues were shuffled during training and testing. Each batch consists of 64 randomly selected tissues. Within this episode loop, the initial state of the simulation was input into the environment and the agent was initiated and started to explore the 2D LA tissue and search for the best ablation technique. The agent moved with a 10-ms step, at each step ablating a small area of 9×9 pixels and then moving into a new position. The ablator in this simulation worked by setting the diffusion coefficient to 0 in the small ablated area. Similarly, an agent was trained using a 50-ms ablation interval to evaluate the effect of the speed of ablation.

At first, the agent moved randomly ablating at each step, receiving positive or negative rewards. With time, the agent learned to predict that the most beneficial moves maximise the cumulative reward, which due to the nature of the reward structure, was achieved when AF was terminated. At each time step, the current state – the agent’s position, the action it made, the corresponding reward acquired – as well as the new state after the move was completed and whether it was a terminal state, were stored. This process is schematically illustrated in Figure 3. The success rate was stored and compared to previous success rates every 50 episodes, and if the success rate was higher than any previous one, the algorithm saved the Q-values corresponding to this reward structure, which was later used in testing on an unseen set of 2D LA tissues.

### Q-Learning Reward Structure

To learn how to predict the value of actions, the agent needs an assignment of numerical values to sets of states, which is called as the reward policy. This acts as a ground truth from the perspective of the agent and is therefore essential in this algorithm. The first reward to be implemented was a positive reward if the ablation strategy was successful. This was defined by rotor termination when the voltage at every point in the tissue was lower than a threshold of 0.2. This would mean that the episode was completed, and the agent had successfully terminated the rotor, rewarding the agent with +420 points, the highest reward possible.

On the other hand, there was also a negative reward, which was implemented every time the agent took a step. This was done to avoid too much scarring of the tissue, making the agent look for the fastest way of terminating AF; the episode was aborted if too much tissue was ablated. This was implemented by calculating the percentage of tissue being ablated and stopping the episode if it reached a rate of 40% ablation of healthy tissue, giving the agent −50 reward and terminating the episode. Moreover, the agent was given a negative reward each time, and it took a step to make it prioritise faster routes to a successful ablation, further avoiding destroying healthy tissue. The agent was also given a negative reward for stepping on the same tissue it had already ablated. The reward structure was further enhanced to prevent the agent from going in a straight line as that was the easiest way to stop a rotor by creating an obstacle between two boundaries of the 2D tissue. This was done by giving the agent an exponentially growing negative reward the more consecutive moves in the same direction it made.

Rotor tip locations were calculated at each ablation time step, and the agent was rewarded for moving closer to the rotor tip and was given a negative reward for moving away from it. Pre-determined successful lesions (such as PVI and fibrosis-based ablation) have been obtained for the 2D LA tissues (Muffoletto et al., 2021) and used to train the agent, giving it a reward of 15 for moving closer to these lesions and −15 for moving further away from them. All the rewards used to train the agent are summarised in Table 1.

**Table 1:** Summary of the reward structure, how many points the agent receives as a reward and whether it is a terminal state.

Reward structure		
Action	Points rewarded	Terminal state
Successful CA – the voltage at every point is lower than 0.2	420	Yes
40% of healthy tissue is ablated	−50	Yes
80 steps have been taken by the agent	−50	Yes
At each step taken	−1	No
Ablating already ablated tissue	−5	No
Moving in the same direction	0.01*exp(*N*), where *N* is number of movements in the same direction	No
Moving closer to the rotor tip	15	No
Moving further away from rotor tip	−15	No
Moving closer to pre-determined successful CA lesion	15	No
Moving further away from pre-determined CA lesions	−15	No

### Deep Learning Networks

Two identical CNNs were created to ensure stable predictions for the Q-values of the available actions. These were both initialised with the same weights. This was done to have a time delayed version of the network for prediction, while the other network was trained. After five episodes the predicting network was updated to share the same parameters as the trained network. This was done with the purpose of increasing stability in the network and to create a classifier, which avoids overfitting. To select an action for the agent to take, the state consisting of 150×150 RGB image was observed. This state was recorded and passed through the CNN.

Both CNNs were built using the Keras Sequential package in Python, starting with a 150×150×3 input layer using separate RGB channels, the input being the 2D LA tissue with the initiated rotor wave. The input is connected to a 2D convolutional layer with 3×3 kernel size and a dimensionality of the output space equal to 256, which signifies the number of output filters in the convolution. A 2D maximum pooling layer (with the pooling layer size of 2×2) was then added in order to down sample the input and only take into account the maximum values. A dropout layer of 0.2 was then added to subsample the input and avoid overfitting.

All the layers mentioned above, starting from the 2D convolution layer, were then repeated and flattened and densed in order to obtain a 1D feature map from the 3D input. Finally, the output of this was put through another dense layer with the size of the action space and a linear activation function. A mean square error loss function and an ADAM optimiser were used.

ɛ-greedy action selection policy was used to choose an action. This action selection policy employs a global parameter ɛ, which defines the probability that the action with the highest predicted Q-value is chosen. In the case, it is not chosen, the selected action will be uniformly sampled from the other available actions. During training, *ɛ* is decayed, which serves the purpose of defining a balance between an exploration vs. exploitation regime. Once the action has been chosen, the agent alters the environment by ablating the underlying tissue. Subsequently, the next state is observed and the cycle repeats.

Based on the reward structure and the Q-values, the agent learns to better predict the value of actions, which allows it to move in the best possible direction, avoiding negative rewards. This could be compared to losing points in a game. This network is able to distinguish different features in the environment, for example, where the wave currently is and how it is moving, as well as the areas of fibrosis, making the agent’s movements more informed.

## Results

### Q-Learning Algorithm Training

**Figure 4:**
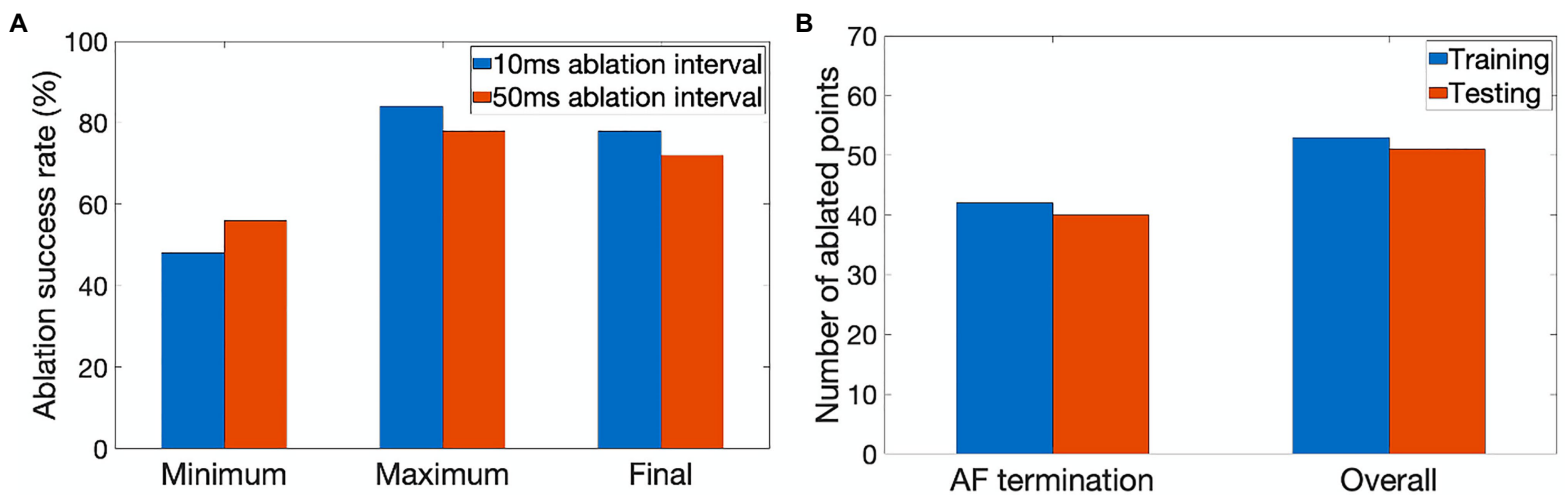
Performance characteristics of the ablating agent. **(A)** The minimum, maximum and final ablation success rate achieved during training for 10ms (blue) and 50ms (orange) ablation intervals. **(B)** Average number of ablated points (9×9 pixel lesions) in cases when AF was terminated successfully and the overall number of cases during training (blue) and testing (orange).

The agent was trained for 900 episodes, exploring the environment of 2D atrial tissues with AF and learning the ablation strategies that provided the highest reward. During training, the success rate was stored every 50 episodes for which the minimum, maximum, and final success rate per 50 episodes can be seen in Figure 4A. The agent rapidly improved in the first few episodes, then fluctuated and settled around episode 500 at approximately 78% success.

**Figure 5:**
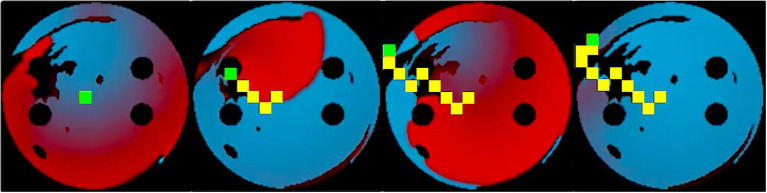
Training sequence at 10ms ablation interval with the ablator (green) successfully ablating the rotor by following its tip. Same colour code is used for the voltage maps as in Figure 2. Ablated tissue is shown by 9×9 pixel yellow rectangles.

The success rate in this case signifies the percentage of successfully terminated rotors per 50 episodes. The highest success rate per 50 episodes the agent was able to achieve after training for 900 episodes was 84%. The model which achieved the highest success rate was then used for testing. Figure 5 shows the agent successfully being able to ablate the tissue using an equivalent of the rotor technique – trying to follow the tip of the rotor in order to terminate it during training. The ablator was rewarded for trying to minimise proximity to the rotor tip.

### Q-Learning Algorithm Testing

**Figure 6:**
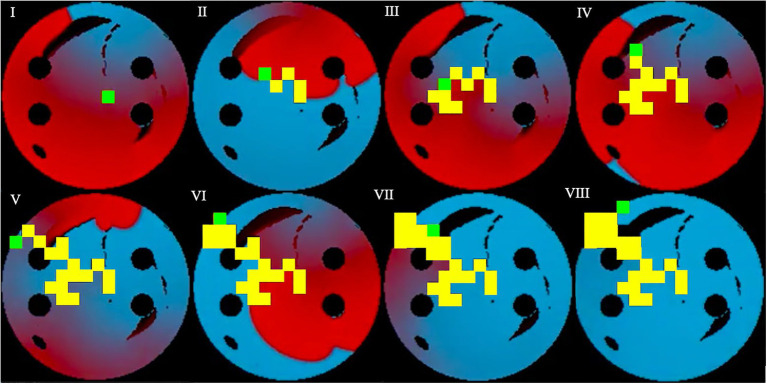
Testing sequence, where the ablator (green) uses the pre-existing model and Q-values from training to successfully terminate the rotor. Same colour code is used as in Figure 5. Voltage distributions in consecutive moments of time are shown in **(I)–(VIII)**.

During testing, the process was similar to training, except the known ablation strategies and, the rotor tip positions were not included in the state passed to the agent, and the most successful network determined in training was used. In this case, for the 10-ms ablation interval, the model with 84% success was used. After testing the model on 100 unseen 2D tissues for 100 episodes, the testing success rate was at 72%. The slight decrease of success rate in testing was expected, since in training the ablating agent was rewarded for moving close to the successful ablation lesions known from earlier simulations – whereas during testing the agent had no information on the location of such lesions, and hence was operating in a more difficult environment. Note also that overfitting in this case is highly unlikely, given the huge number of possible AF scenarios (i.e., movements of one or more re-entrant waves over greatly variable trajectories in a large number of LA tissue models with different spatial characteristics). Figure 6 shows the agent successfully terminating the rotor during a test. During testing, all the ablation points for each 2D tissue model were saved to be used later in the respective AF recurrence check.

Figure 4B quantifies the tissue damage during ablation. As expected, the average number of ablated points in cases when AF was terminated successfully was lower than in the overall number of cases. The number of ablated points was about the same in training and testing.

### Recurrence Testing

The ablation strategies developed by the Q-learning algorithm were also tested for success in preventing AF recurrence, specifically to check whether the existing ablation lesions would prevent the rotors from restarting. This was done by attempting to initiate four different rotors in 2D atrial tissue models after ablation (with the non-conductive CA lesions present) and to check whether the lesions saved in testing would stop the rotors, and thus prevent the recurrence. Note that this test did not involve any Q-learning process, but only LA model simulations.

**Figure 7:**
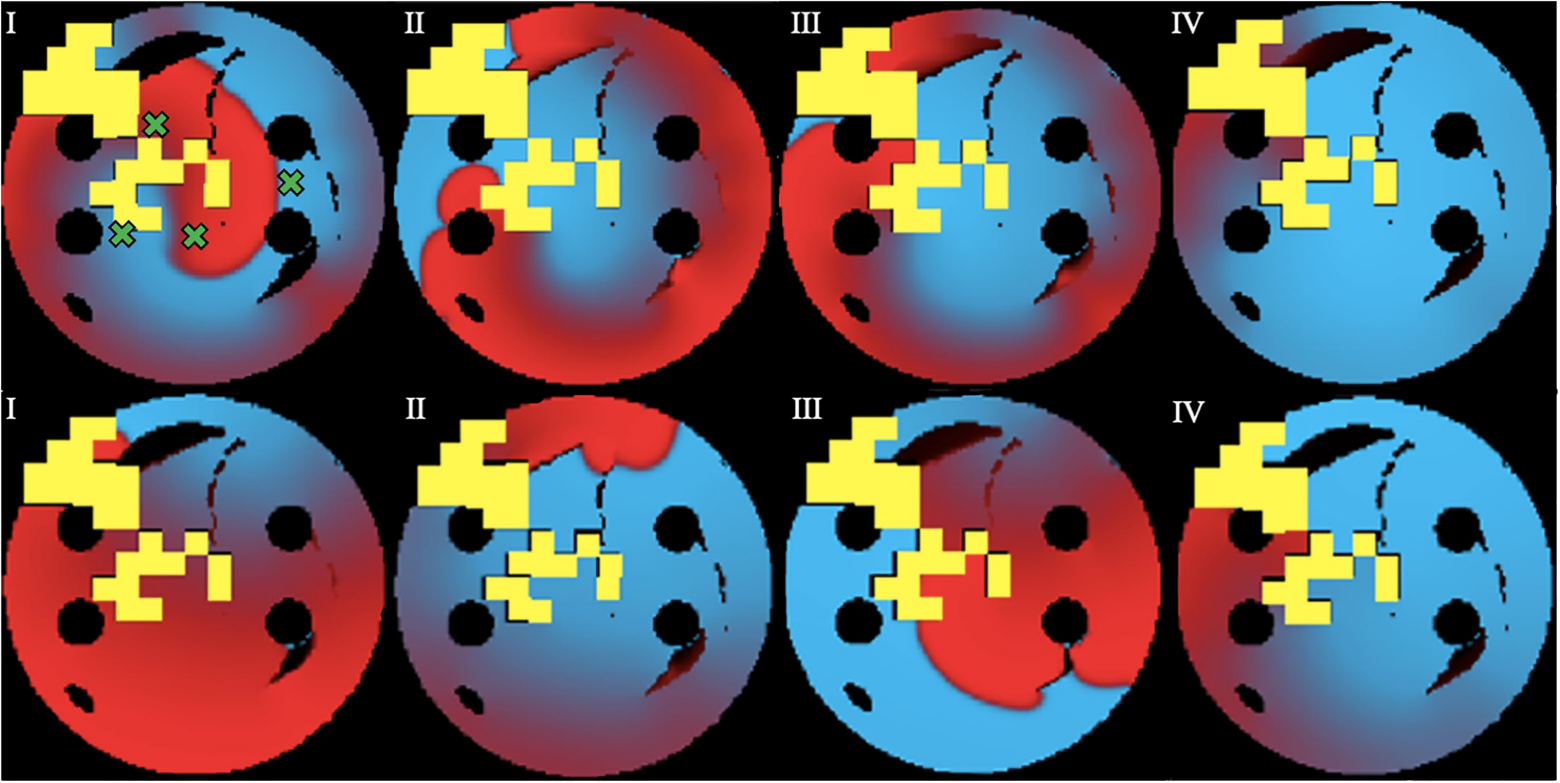
Prevention of AF recurrence is shown using the successful ablation strategy identified by the agent during testing. This happens when rotor is re-initiated with both Scenario 1 (top row) and Scenario 2 (bottom row). Green crosses show the four locations of rotor initiation; the locations were chosen to produce most stable rotors pre-ablation. Voltage distributions in consecutive moments of time are shown in **(I)–(IV)**.

In Figure 7, successful ablation lesions created by the agent during the testing can be seen preventing the newly initiated rotor from propagating (Scenario 1), meaning that this AF scenario was not sustained. To check this result was independent of the initial rotor location, another scenario for the rotor initiation was tested: again, the rotor also was not sustained (Scenario 2). Simulations were also repeated for two more rotor locations (Scenarios 3 and 4, not shown). In all four scenarios, the rotors failed to sustain AF in most 2D LA tissues. Specifically, the success rate of recurrence testing was 98% for a single scenario and 89% for all four scenarios. This means that rotors were terminated by ablation patterns determined by the Q-learning agent during testing in 89% of the cases.

## Discussion

This study shows that Reinforcement Q-Learning algorithms supported by CNNs can predict patient-specific ablation strategies that are effective in both terminating AF and preventing its recurrence in LGE-MRI-based 2D LA tissue models. This was achieved by simulating AF scenarios in 2D atrial models and using them as the environment for a Q-learning algorithm. The algorithm was further tested on an unseen set of 2D LA tissue models by using the most successful version of the Q-learning network, as well as by using ablation lesions produced during the testing to check for the likelihood of AF recurrence in these models. The ablation success rate was 84% in training and 72% in testing (at 10-ms ablation interval), showing that the agent explored the environment and learned to ablate successfully, and that Q-learning can be a viable method to improve CA strategies for patient-specific AF cases. Furthermore, the overall recurrence prevention success over 89% (i.e., 11% recurrence), surpassing that of existing CA methods, which resulted in about 55–80% recurrence rates (Dretzke et al., 2020).

**Figure 8:**
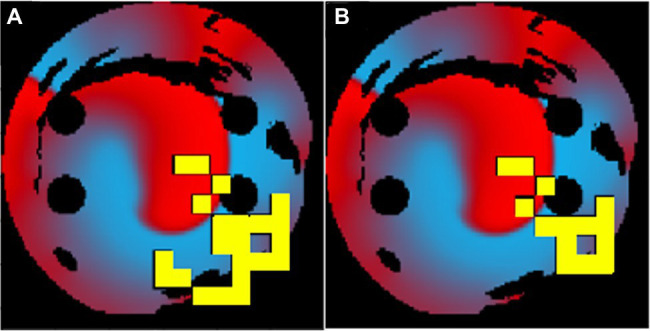
The same patient-specific 2D LA tissue models ablation performed by the agent at **(A)** 10ms interval and **(B)** 50ms interval. The ablation patterns are similar but not identical.

When a longer 50-ms ablation interval is used, the ablation patterns were similar to those of the 10-ms ablation interval for the same tissues. As seen in Figure 8, the 50-ms interval ablation points can be found in the same positions for the 10-ms interval ablations but less tissue was ablated. The main difference between the two cases was the total ablation time, and since the 10-ms ablation interval ablates more frequently, it has additional ablation points added, before AF is terminated. This suggests that the Q-learning algorithm prioritised the atrial tissue structure (such as location of fibrosis) and function (location of the wave at the time of ablation) over the ablation interval. This implies that simulations do not necessarily have to be run for long periods of time in order to find the optimal patient-specific ablation pattern. In effect, shorter simulations could be run, and computational expense could be reduced.

Note also that the action space of the ablating agent in this study was discrete: the agent could only choose a set of discrete actions to perform. This may be more limited than using a continuous action space, in which the agent can perform a continuous set of actions – in our study, a continuous range of movements in the LA tissue. However, when comparing continuous vs. discrete deep reinforcement learning algorithms, it has been shown that with a limited number of trials the discretised approach outperforms the continuous one (Smart and Laelbling, 2000; Stopforth and Moodley, 2019). Moreover, the action space is discretised to enable the use of a deep Q-learning network, which requires a discrete action space as the size of the output layer of the network corresponds with the size of the action space.

One limitation of the approach used in the current study is that the Q-learning algorithm appeared to learn less from pre-determined successful ablation strategies (such as PVI), mostly preferring the “rotor” strategy aimed at ablating the tip of the rotor, similar to previous research (Muffoletto et al., 2021) during which a classifier was trained to find the best ablation strategy and found the “rotor” was the preferred strategy. Such information, however, will not be available for real patients. Given more time for training, the algorithm could further improve and learn more information from the tissues. However, this was hindered by significant computational expense, making it impractical to train on a large number of tissues if the ablation interval is long. Moreover, the reward structure could still be improved, as the ablator does not always take the shortest path, which can be seen in Figure 6, and often tries to ablate previously ablated tissue. Ideally the agent should move through the shortest path possible.

To improve this work in future, the computational load should be minimised, as currently the 10-ms ablation interval technique takes 30min to run per episode while a 50-ms ablation interval takes about 5h 30min per episode. Using a similar approach in a clinical setting will require the application of GPU to accelerate simulation time. Furthermore, a more clinically relevant approach will require patient-specific 3D atrial models instead of 2D models as input into the Q-learning algorithm to produce more accurate results.

Data augmentation techniques could also be applied to enhance the patient datasets. In the previous study (Muffoletto et al., 2021), 122 real patient-specific LA images have been used to create additional synthetic images by taking random weighted averages of all the real data set to introduce new fibrotic patterns, and also varying size of the PVs. In the current study, 50 such synthetic images were used for additional testing, with the success rate remaining at 72%.

Machine learning has been applied in this field before, thus Lozoya et al. (2019) have achieved 97.2% accuracy in finding ablation targets using biophysical cardiac electrophysiology models to augment ventricular image features. However, their feature augmentation algorithm used supervised learning, whereas Q-learning is neither supervised nor unsupervised. A study by Liu et al. (2020) used computed tomography images of the atria to train a CNN and create a prediction model of the non-PV triggers for AF, reaching an accuracy of 82%. However, instead of predicting ablation techniques, their method simply identified patients with a high risk of non-PV triggers.

Building upon the recent advancements in applications of deep learning in cardiac imaging and modelling, our study provides a unique approach to tackle the problem of AF ablation leading to recurrence. The developed approach could be translated to the clinic, with routine LGE MRI scans used to create patient-specific LA models and the trained Q-learning algorithm then applied to predict a suitable ablation strategy for the patient. The predicted pattern can then integrated into the ablation image-guidance system to provide additional information for cardiologists performing the ablation procedure. Thus, after further clinical validation, our proof-of-concept Q-learning technique can be applied both to improve understanding of patient-specific ablation therapy and to enhance current clinical treatment methods.

## Data Availability Statement

The original contributions presented in the study are included in the article/supplementary material, further inquiries can be directed to the corresponding author.

## Author Contributions

AB, MM, and OA conceived and designed the study. LM, AB, and MM substantially contributed to computations and data analysis. OA contributed to interpretation of the results. AQ, AZ, and AR contributed to data analysis and computations. LM and OA drafted the manuscript. LM, AB, AQ, AZ, AR, MM, and OA edited the final version. All authors have made significant contributions to this study, and also approved the final version to be published, while agreeing to be accountable for all aspects of the work in ensuring that questions related to the accuracy or integrity of any part of the work are appropriately investigated and resolved.

## Conflict of Interest

The authors declare that the research was conducted in the absence of any commercial or financial relationships that could be construed as a potential conflict of interest.

## Publisher’s Note

All claims expressed in this article are solely those of the authors and do not necessarily represent those of their affiliated organizations, or those of the publisher, the editors and the reviewers. Any product that may be evaluated in this article, or claim that may be made by its manufacturer, is not guaranteed or endorsed by the publisher.
