# Corrigendum: Reinforcement Learning to Improve Image-Guidance of Ablation Therapy for Atrial Fibrillation

**DOI:** 10.3389/fphys.2021.799585

**Published:** 2021-11-09

**Authors:** Laila Muizniece, Adrian Bertagnoli, Ahmed Qureshi, Aya Zeidan, Aditi Roy, Marica Muffoletto, Oleg Aslanidi

**Affiliations:** ^1^School of Biomedical Engineering & Imaging Sciences, King's College London, London, United Kingdom; ^2^Department of Biomedical Engineering, ETHZürich, Zurich, Switzerland; ^3^Department of Computer Science, University of Oxford, Oxford, United Kingdom

**Keywords:** atrial fibrillation, catheter ablation, patient imaging, reinforcement learning, deep learning

There is an error in the Funding statement. The correct number for ^**^British Heart Foundation^**^ is ^**^PG/15/8/31130^**^.

The authors apologize for this error and state that this does not change the scientific conclusions of the article in any way. The original article has been updated.

## Publisher's Note

All claims expressed in this article are solely those of the authors and do not necessarily represent those of their affiliated organizations, or those of the publisher, the editors and the reviewers. Any product that may be evaluated in this article, or claim that may be made by its manufacturer, is not guaranteed or endorsed by the publisher.

